# Practitioner perspectives on the use of acceptance and commitment therapy for bereavement support: a qualitative study

**DOI:** 10.1186/s12904-024-01390-x

**Published:** 2024-02-28

**Authors:** Nikolaus Willi, Anna Pancoast, Ioanna Drikaki, Xueying Gu, David Gillanders, Anne Finucane

**Affiliations:** 1 Clinical Psychology, University of Edinburgh, Edinburgh, UK; 2 Marie Curie Hospice Edinburgh, Edinburgh, UK

**Keywords:** Acceptance and commitment therapy, ACT, Bereavement, Grief, Qualitative research, Psychological adaptation, Coping skills, Coping behaviour, Coping strategies, Psychological well-being

## Abstract

**Background:**

There is currently a high demand for bereavement support coupled with inconclusive findings as to the efficacy of existing approaches. Acceptance and Commitment Therapy (ACT) aims to improve human functioning and has shown efficacy across a wide range of conditions. ACT may be a promising means of supporting bereaved people, yet evidence on the use of ACT for bereavement support is lacking. The aim of this study is to explore how ACT is currently used for bereavement support and practitioner perspectives of how it helps following bereavement.

**Methods:**

Semi-structured interviews were conducted online via MS Teams with practitioners experienced in using ACT for bereavement support. Data were analysed thematically guided by a framework approach.

**Results:**

Nine participants were recruited. Three themes were identified: (i) creating psychological space around grief; (ii) using psychological space for value-directed action in the midst of grieving, and (iii) adapting ACT for bereavement support. Practitioners indicated that ACT improves clients’ relationship with distressing internal experiences. Metaphors and mindfulness techniques were used to encourage acceptance of grief responses, taking perspective on distressing thoughts and images, and contact with the present moment. Better relationships with distressing experiences were regarded as less psychologically taxing, improving coping and well-being, while providing the psychological space to engage in value-directed action. Values exploration, sometimes using metaphors and exercises, was seen as supporting the bereaved person to rediscover a sense of purpose and engage in meaningful activities alongside their grief. Practitioners used ACT flexibly, integrating other interventions, and adapted ACT to the perceived sensitivities of bereaved people, and age-related and developmental factors.

**Conclusion:**

ACT is used to support people who have been bereaved to live effectively with the difficult thoughts and feelings associated with grieving and to enable them to gradually identify, reconnect with, and act in line with their values after loss.

**Supplementary Information:**

The online version contains supplementary material available at 10.1186/s12904-024-01390-x.

## Background

Grieving involves a range of powerful cognitive, emotional, and behavioural disturbances that typically last between 6 and 12 months but can endure significantly longer [1, 2]. These may include intense yearning, sadness, guilt, anger, loneliness, and disbelief; ruminative thoughts and memories; loss of motivation, meaning and purpose; and disengagement from social, leisure, and other valued activities [3, 4]. Consequently, grief is often accompanied by significant distress and dysfunction [5]. Generally, grief becomes less intense over time: ruminations and emotions diminish in frequency and intensity, the reality of the loss is accepted, and a renewed sense of purpose is developed [6]. While grieving continues, it becomes integrated with life [7]. The grieving period varies by individual and is influenced by factors such as the relationship with the deceased, the age and cause of death, and culture (e.g. 8–10). However, some individuals experience difficulties in the integration of loss. An estimated 7–10% of individuals develop prolonged grief disorder [11, 12], which describes the persistence of intense grief and associated symptoms severe enough to cause problems in the person’s life [13].

Approximately 40% of people may benefit from formal bereavement support provided by trained volunteers, health and social care professionals, or mental health specialists, including therapists, psychologists, and counsellors [14, 15]. Yet in a survey of bereaved people (*N* = 2189) conducted in the United Kingdom (UK) in 2019, only 9% received support of any kind and, of those, only 35% found it helpful [16]. Moreover, a recent assessment of UK bereavement support found it to be highly varied and lacking in evidence for its effectiveness [17]. Bereaved people are at increased risk of prolonged grief disorder [12], other psychological disorders [18], and suicide [4]. While only a minority develop psychological disorders and suicide is rare, the severity of such outcomes coupled with the intense distress and dysfunction associated with grieving call for timely access to effective bereavement support.

**Fig. 1:**
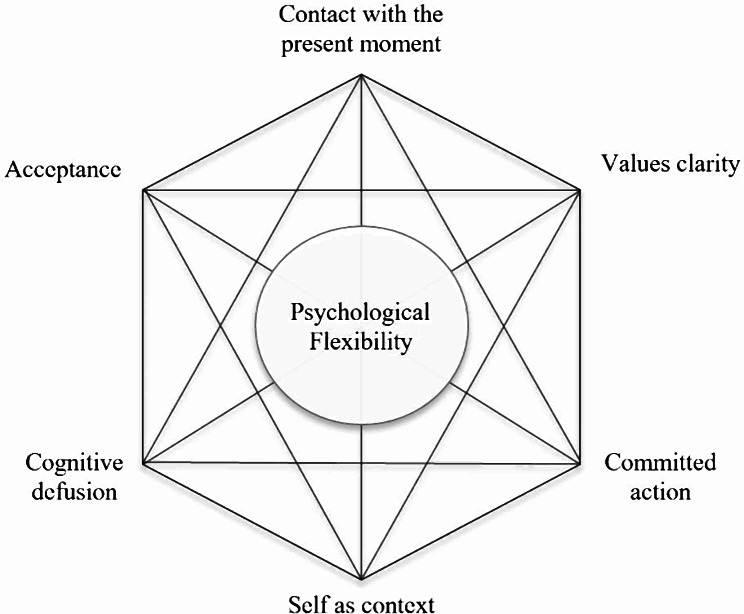
The ACT “Hexaflex”

Acceptance and Commitment Therapy (ACT, said as one word rather than three letters) is a form of cognitive behavioural therapy typically covered by health insurance in the United States of America (USA) and provided by competent practitioners in the United Kingdom’s (UK) free-to-access National Health Service. It is theoretically well-suited to improving coping and well-being following bereavement. The aim of ACT is to increase psychological flexibility by promoting acceptance of unwanted inner experiences and commitment to behaviour change in a value-directed way [19]. Psychological flexibility is the ability to locate oneself in the present moment with full awareness, while adapting one’s behaviours towards valued ends regardless of the presence of distressing experiences [18, 20]. Psychological flexibility comprises six overlapping and interdependent processes (21; Fig. 1).

Acceptance is embracing inner experiences (such as feelings and thoughts) as they are. Acceptance does not mean people have to like their painful experiences. It is a willingness to contact inner experiences with a more welcoming stance instead of fighting or avoiding them [22]. Avoidance of unwanted thoughts and feelings has been shown to have negative effects on functioning [21]. Cognitive defusion is the process of unhooking attention from the content of distressing thoughts and images and observing them for what they are – thoughts – rather than being excessively reactive to their content. Contact with the present moment is the ability to pay attention flexibly to any current experience rather than being unwittingly absorbed by them, such as rumination about the past or worry about the future [22]. Self-as-context is the recognition of the self as the observer of experience rather than its content [23]. This is sometimes referred to as the ‘noticing self’ or ‘observing self’ – the aspect of ourselves that is aware of what we are thinking, feeling, sensing, or doing in the moment. It can also refer to flexible perspective taking – the ability to perceive events from alternative points of view (e.g. that of a partner, close friend, ‘inner child’, or ‘future self’). Values clarity is being able to recognise what matters most on an ongoing basis and using values to motivate and guide our behaviour. They are different from goals, as goals are obtainable while values are qualities of ongoing action (e.g. acting with honesty, kindness, or creativity) [23]. Committed action is doing what needs to be done to live by our values, even when that brings up difficult thoughts or feelings. This includes physical action (explicit action) and psychological action (how we manage thoughts and feelings).

Acceptance, defusion, contact with the present moment, and self-as-context constitute the mindfulness processes [24]. ACT engages these processes through an array of metaphorical and mindfulness exercises [25]. For example, paying close attention to thoughts and feelings without resistance, and taking perspective from them by imagining that they are like clouds in the sky, coming and going, and sometimes returning [26]. ACT fosters values clarity and committed action through self-exploration and goal-setting exercises. For people who have been bereaved, these processes may improve their ability to cope with intense grief-related emotions and rumination, while processes linked with clarifying values and committed action may help with issues such as a loss of motivation, meaning, and purpose, and disengagement from activities.

ACT fits well with two prominent theories of grieving – the Dual Process Model [27] and Continuing Bonds [28]. According to the Dual Process Model there are loss- and restoration-related bereavement stressors. Loss-oriented coping involves the processing of events leading up to the death, accepting its finality, and confronting intrusive thoughts of the deceased. Restoration-oriented coping involves attending to changes caused by the death, engaging in new activities, and establishing a revised identity and social role. Bereaved individuals cope by oscillating between these orientations. Continuing bonds with the deceased through an ongoing inner relationship may also improve coping while grieving [29], for example, by reminiscing, keeping photographs and possessions, or by retaining the deceased’s values internally and expressing them through action [30]. ACT’s focus on contacting the present moment, accepting unwanted inner experiences, and committing to value-directed behaviour change may facilitate the movement between loss-oriented and restoration-oriented processes by helping individuals track what is influencing them and make more conscious choices about how they deal with situations, and the thoughts and emotions that they occasion. Moreover, this focus may also help them to engage with the memory of the deceased and bring it into the future with them (continuing bonds). Despite its potential, research on the use of ACT for bereavement support is lacking. A recent systematic review identified only one published paper [31] and one doctoral thesis [32] focused on its use for bereavement support [33]. The former is a randomised control trial involving 106 bereaved carers assigned to an ACT intervention or control group [31]. The intervention – an ACT self-help booklet and telephone support – was feasible and acceptable to participants, though data attrition was high, and effect-sizes were small and not statistically significant. The doctoral study used interpretative phenomenological analysis to explore experiences of two bereaved spouses and six therapists who had used ACT for bereaved support. The bereaved spouses found that ACT helped them deal effectively with undesirable thoughts and feelings and supported them to identify memories, which facilitated a continuing bond with the deceased and helped them re-engage with life. Therapists found that ACT was useful in helping normalise grief as opposed to problematising it, and all reported positive experiences with using ACT for bereavement support.

## Aims

Given the dearth of research, further exploratory work is needed as a first step to understanding how ACT is used in practice for bereavement support. Specifically, we aimed to answer the following questions from the perspective of practitioners:

- How is ACT used for bereavement support in practice?
- Which ACT processes are perceived by practitioners as core to bereavement support?
- How does ACT lead to improvements in coping and well-being following bereavement?

The findings will inform the development of ACT-based interventions to improve coping and well-being for people struggling with grief and loss.

## Methods

### Design

A qualitative study involving online semi-structured interviews conducted via MS Teams.

### Participants

We recruited practitioners who use ACT to support clients who have been bereaved. To be eligible, potential participants needed to have had some formal training in ACT; consider themselves competent in delivering ACT; have at least one year of experience using ACT to support bereaved people; and speak English. There was no restriction on the location of participants.

### Participant recruitment

We used convenience sampling. We posted adverts for the study on social media including Twitter and LinkedIn. We also advertised the study on the global, and the UK and Ireland Contextual Behaviour Science Facebook pages and shared information about the study with two hospices in Scotland. Potential participants emailed the research team for further information and received a link to a webpage, which included the participant information sheet and consent form. On completion of the consent form, participants were asked to complete a short questionnaire to provide some background demographic information so the sample could be described.

### Data collection

Interviews were conducted online via MS Teams by masters-level postgraduate researchers NW, AP, ID and XG, who received training in qualitative research. Interviews were audio and video recorded and lasted between 25 and 60 min, with an average time of 50 min. To ensure quality and consistency, the interviews were conducted in pairs. One researcher acted as the lead interviewer and the other kept time, tracked the interview schedule, and was permitted to ask participants to elaborate. The pair would then swap roles in the next interview. Interviews were automatically transcribed by MS Teams software and cross-checked and amended against interview audio by one of the researchers. The researchers had no previous relationship with the participants.

### Data analysis

Interview data were analysed by NW, AP, ID and XG using the framework approach [34]. Both inductive and deductive analysis were used. The analysis was conducted in seven stages supported by NVivo 12 Plus coding software [35]. The steps included transcription, data familiarisation, coding, analytic framework development, framework application, charting and interpretation [34]. This approach allowed us to structure the data within the parameters of the research questions while retaining open coding and unanticipated theme generation, which balances relevance and scope. Data were arranged in framework matrices that link data with codes and cases. This facilitated thorough data comparisons within and across cases [34]. For the interview schedule, participant characteristics questionnaire, analytic framework, an example framework matrix, and an early thematic map, see Supplementary Information.

### Reflexivity

Quality was enhanced and researcher bias minimised in several ways. First, we followed the Consolidated Criteria for Reporting Qualitative Research (COREQ; 36) guidelines wherever possible (see limitations). Second, the research team met frequently over a period of six months to reflect on the research process and data. Third, the researchers undertaking data analysis compared and honed their analytic frameworks and then iteratively charted data and refined coding through the framework matrices. These processes increased credibility by triangulating data analysis, maximising its relevance to the data and ensuring thematic saturation [34, 37]. Fourth, the methods section and supplementary information provide an audit trail of decision-making, analytic processes, and materials, which increased transparency [38]. Fifth, a description of the research setting and participants (see Table 1) facilitates the reader’s interpretation of the data [37]. Sixth, each researcher undertaking data collection and analysis kept a reflexive journal, analysing and sharing their subjective responses to the data [39]. Seventh, participants were sent the final draft of the research article for review.

## Results

### Participant characteristics

Nine participants were recruited – four clinical psychologists, three social workers, one counsellor, and one psychological therapist. Most had used ACT for bereavement support for two to three years (range: 1–16 years). All participants self-identified as ethnically white. Six were based in the UK and three were based in the USA.

**Table 1:** Participant Characteristics

Participant	Gender	Age (years)	ACT experience (years)	Also supports CYP*	Region	Practitioner-type
Pierre	Male	25–34	5		UK*	Clinical psychologist
Alba	Female	25–34	3	Yes	UK	Clinical psychologist
Trisha	Female	35–44	1	Yes	UK	Counsellor
Ophelia	Female	25–34	3		USA	Social worker
Angus	Male	45–54	2–3		UK	Social worker
Samantha	Female	45–54	2		UK	Psychological therapist
Stanley	Male	55–64	2	Yes	UK	Clinical psychologist
Danielle	Female	55–64	16		USA	Clinical psychologist
Hanna	Female	35–44	4		USA	Social worker

*Note* names are pseudonyms. *CYP: children and young people

### Overarching themes

**Fig. 2:**
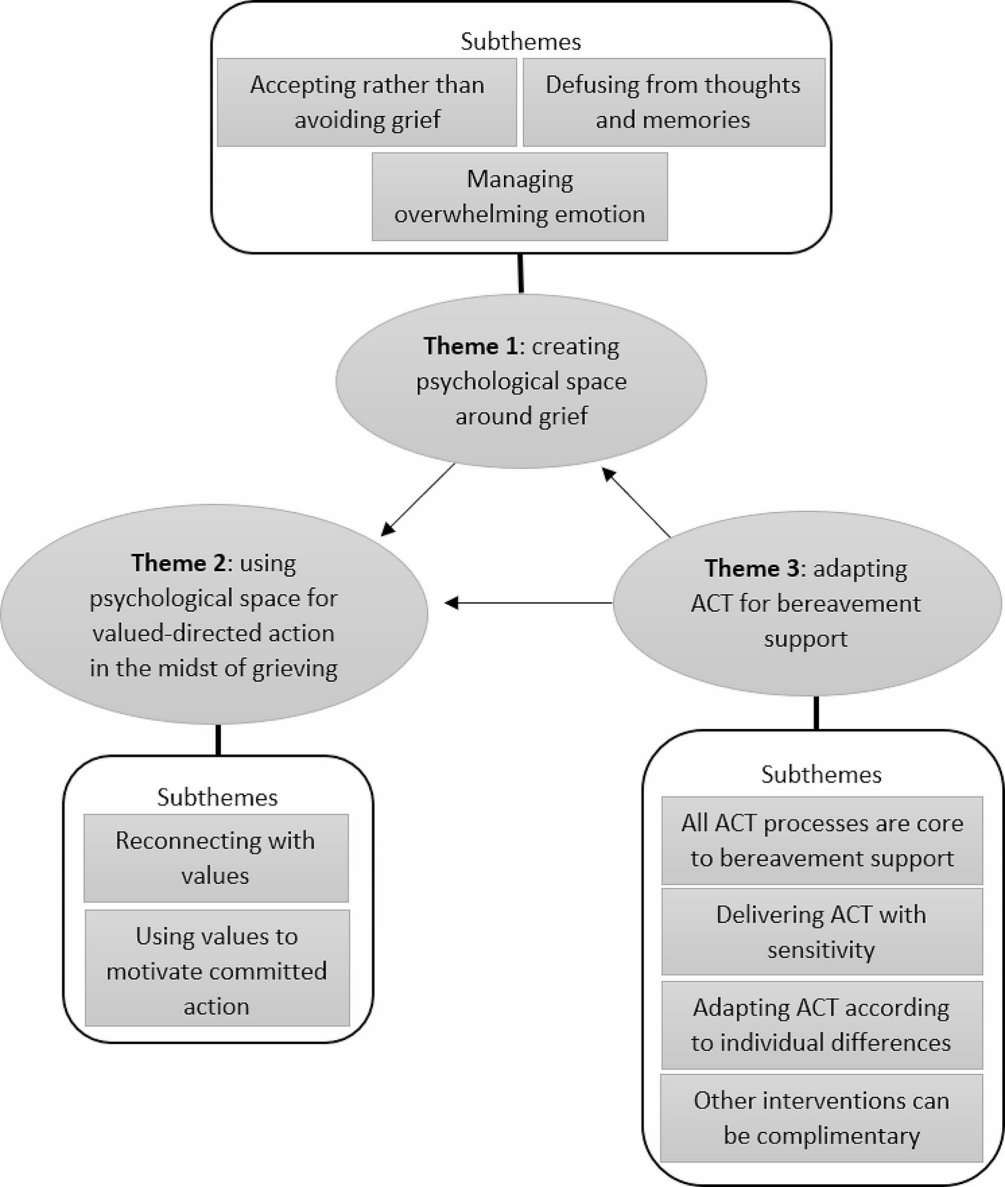
Thematic map illustrating practitioner perspectives on the role of Acceptance and Commitment Therapy for bereavement support. As described in the results below, the arrows represent practitioners’ view that Theme 1 facilitated Theme 2 and Theme 3 facilitated both Theme 1 and 2

Three overarching themes and nine sub-themes (Fig. 2) were identified regarding practitioners’ perspectives on how ACT is used to improve coping and well-being for bereaved people:

1. creating psychological space around grief
2. using psychological space for value-directed action in the midst of grieving
3.  adapting ACT for bereavement support

### Theme 1: creating psychological space around grief

#### Accepting rather than avoiding grief

Practitioners reported clients often tried to avoid distressing thoughts, memories, and emotions, and viewed grief avoidance strategies as disruptive, exhausting, and futile. These strategies included repression of, and distraction from, distressing internal experiences.

*It’s difficult for any of us to have control over our thoughts and feelings. And, in a way, it’s a waste of time. […] The energy around trying to control the thoughts is exhausting.* (Angus)

Avoidance strategies were seen as stalling the grieving process. Practitioners reported that accepting rather than avoiding the presence of grief-related experiences had the dual benefit of reducing the struggle with internal experiences and allowing the grieving process to continue.

*Is the avoidance what prolongs people’s challenge? […] The other theme that comes up for me is that grief wants to be grieved. So, you know, avoidance is not only relatively futile, but it feels counterintuitive.* (Danielle)

Practitioners encouraged clients to stay in contact with distressing internal experiences and allow grieving by facilitating their willingness to feel loss and pain. Clients were often perceived as naïve regarding grief, failing to realise the extent of its psychological and physiological consequences. According to practitioners, they were thereby also unable to articulate their experiences, which increased fear and the sense of being overwhelmed. Acceptance was facilitated by promoting literal, metaphorical, and experiential understanding of grief through psychoeducation and mindfulness exercises. For example, Alba and Samantha used a metaphor which describes suffering and value as two sides of a coin: people suffer only because something they value is at stake.

*We could just […] chuck [the coin] away; get rid of it. But then what are you left with? So, you get rid of the sadness and the pain, but you’re left without the sweet moments of care and love you had with that person. […] Most people say ‘You know what? I’m gonna keep it’.* (Samantha)

Mindfulness techniques provided experiential understanding by encouraging clients to notice and become familiar with mental and physiological experiences related to grief.

*I think there’s something about working with people at the beginning to, kind of, accept what grief maybe is and how it’s impacting on them. And a lot of people want to move really quickly away from their grief or their feelings. So, I think a lot of that is trying to get people to notice what they’re feeling in their body. And that is through a lot of mindfulness techniques […] so it feels a bit less scary.* (Trisha)

Mindfulness techniques sometimes had imaginal elements. For example, to help clients identify and stay with an experience, some practitioners encouraged physical visualisation of the grief experience– whereby one imagines it to have a particular shape or colour. At other times, imaginal mindfulness exercises were used to encourage staying in contact with distressing internal experiences through self-compassion.

*It’s a practice to identify […] a difficulty; to find it in a somatosensory way. ‘Does it feel like a kick in the gut? Does it feel like a punch to the throat? Where is it physically in my body?’ To then think of a source of support, compassion, acceptance, and to imagine taking [their hand] – maybe that beloved friend, or that beloved pet, or that nurse that really cares for you – and putting the hand on the stuck or hurt place. And then breathing into that place with a sense of acceptance and space. […] Instead of saying ‘No, I won’t have this feeling’.* (Ophelia)

#### Defusing from thoughts and memories

Practitioners found that clients were often consumed by distressing thoughts and memories relating to their loss, such that they were fused with them. Practitioners regarded fusion as constant preoccupation with the content of thoughts and memories, leaving little or no space for anything else. According to practitioners, clients’ thoughts and memories were often frightening, traumatic, or centred around self-blame. Fusion was viewed as increasing clients’ sense of being overwhelmed and reducing their capacity to be in the present moment and act.

*All of us are struggling with difficult thoughts. [...] But sometimes those thoughts occupy all the space. […] So maybe we can do something to try to create some space in between [the client] and those memories and those ideas when there’s something else that deserves that space.* (Pierre)

Practitioners employed ACT techniques to support clients to defuse, which they viewed as creating distance, or perspective, from these thoughts and memories while still allowing them to be present. The content of thoughts and memories was not challenged; rather, they were no longer seen as necessarily demanding attention. Taking perspective was thought to diminish distress by reducing the struggle with, and the sense of being overwhelmed by, distressing thoughts and memories, allowing clients to reconnect with the present moment.

Defusion was facilitated through psychoeducation, as well as metaphorical and mindfulness exercises. Metaphors often served the dual purpose of explaining fusion and how to defuse. For example, Angus would use the ACT clipboard metaphor (Harris, 2019). Clients were asked to hold a clipboard vertically in front of their face. Angus would then gently push on the back of the clipboard which caused the clients to resist. When asked what they could see, clients usually responded ‘nothing’. When asked how they felt, clients usually said they were tired from holding the clipboard and struggling against Angus’s gentle push.

*This technique is one way of helping people live with those distressing images and any thoughts around it. So, if you imagine [the distressing thoughts and images are] what your clipboard represents and then, when [defusion] happens, if you just imagine just putting it down in your lap, so you’re not ignoring it – it’s in front of you. What that enables you to do, then, is to see beyond that and connect with the world.* (Angus)

Mindfulness techniques were also used to generate perspective and were often explained with metaphors. Clients were encouraged to pay close attention to thoughts and memories, giving them a sense of being an observer separated from their content. For example, Trisha would ask clients to imagine their thoughts flowing by on water.

*I think that [it] can sometimes take the fear […] or the power out of the thought. And it can give space for somebody, almost […] a bit of respite from the thinking, if you can distance the thoughts […] so you’re not avoiding them, but you feel like you’ve got some power over [them]. Like just watching them instead, rather than feeling like they’re consuming you.* (Trisha)

Practitioners reported that several ACT processes were involved in this kind of exercise, including present moment awareness (paying attention to thoughts and memories), acceptance (not struggling with them), defusion (you are separate from your thoughts and feelings, even difficult ones) and self-as-context (regarding the self as the observer or container of thought, not the content).

*Some people will be really distressed by overwhelming rumination and difficult thoughts. And so, I’ll start on defusion and [the mindfulness processes].* (Ophelia)

#### Managing overwhelming emotion

Practitioners also used ACT to support clients in reducing overwhelm in the face of distressing emotions. Practitioners used the same methods as those used to encourage willingness to grieve (acceptance) and defusion from distressing thoughts and memories; namely staying in contact with emotions but with perspective, so as not to avoid them or be consumed by them.

*[My client] also struggled with, like, not wanting to have these less than pleasant feelings. […] And both defusion and acceptance are really helpful for them. […] We used […] techniques like ‘noticing that you’re having this feeling’ and ‘giving that feeling a little bit of space’, so that it’s not so all consuming.* (Hanna)

*I think learning […] the mindful observation skills of helping people learn how to emotion surf, of how to get some distance […] from the surge of overwhelming emotions […] is really helpful*. (Samantha)

### Theme 2: using psychological space for value-directed action in the midst of grieving

#### Reconnecting with values

According to practitioners, a key element of ACT for bereavement support is helping people explicitly identify their values and then motivate behaviour or action that is aligned with these values. Practitioners suggested there were two obstacles to clients’ engagement with value-directed action. First, as clients’ values were often entwined with the deceased, the loss of a loved one could entail the loss of shared purpose, meaning, and identity.

*When someone dies […] it questions our existence, our purpose. And so, we routinely are with people who are asking themselves the question ‘Why am I getting up today?’.* (Angus)

Second, overwhelming emotion, fusion with thoughts and memories, and avoidance were viewed as consuming, exhausting, and taking up the psychological space needed for action. To improve coping, ACT was used to generate acceptance of, and perspective from, distressing internal experiences. As this creates psychological space around grief, practitioners claimed it provided the capacity for action.

*Anything that helps people sit in the present – even if it’s painful – then gives them the freedom to kind of choose what else they do.* (Alba)

Practitioners sought to support clients to explicitly identify their values through gentle questioning (e.g. asking ‘What is important to you?’) and exercises. The exercises were either fictitious scenarios or worksheet inventories designed to help clients think about their values in different areas of life – such as relationships, leisure, personal growth, and work – and how these informed past actions and goals. For example, Ophelia used an ACT exercise designed to help people elicit their values by taking perspective on their lives:

…*let’s say: ‘In 20 years you’ve grown tremendously from the loss of your loved one. You’ve lived this really full, tremendous life that you were previously afraid to live. You’re at a party and someone says a speech about you. What do they say?’* (Ophelia).

### Using values to motivate committed action

Connecting with values was perceived as helpful in motivating committed action, providing the energy and purpose to move forward after bereavement:

*The values-based work is hugely important because whatever path they choose after bereavement, it will be painful. So, you’ve got to have a really strong sense of your values and who you are to help you do those things.* (Alba)

### Theme 3: adapting ACT for bereavement support

#### All ACT processes are core to bereavement support

Practitioners viewed ACT as targeting not the nature of distressing internal experiences but their clients’ relationship with them: their avoidance of grief, their fusion with thoughts and memories, and their overwhelm in the face of emotion. Practitioners reported ACT improves clients’ coping and well-being by helping them to accept the presence of distressing internal experiences, while taking perspective from them, which reduces their struggle with these experiences. Practitioners also addressed the lack of meaning, purpose, and identity by reconnecting clients with their values, encouraging them to take committed action within the newfound psychological space.

*I think the foundation of ACT and bereavement care is generating and engendering the capacity to hold difficult experiences of loss [so] that they are neither, like, consuming the person, nor are they avoided by the person […] And I think the, you know, ACT without the life worth living piece […] is really just like a stunted, or like a half-ACT. So, I try to do both.* (Ophelia)

All the ACT flexibility processes were perceived as core to bereavement support. The mindfulness processes – present moment awareness, acceptance, defusion, and self-as-context – were all regarded as contributing to the generation of psychological space. On the other hand, practitioners viewed values clarity as generating the motivation to take action which is meaningful to the client (committed action).

*I feel they’re all core. I feel pretty strongly that they’re so interrelated*. (Hanna)

Practitioners did not use a protocol for bereavement support. They assessed their clients’ most pressing needs and addressed those first. However, even if they began with a focus on a specific process, they often felt they used the others throughout their sessions.

*And then, of course, with any given client where I start is not where I end, right? We kind of dance around the [flexibility processes], depending on their needs and their progress. So I don’t have a set, you know, protocol.* (Ophelia)

### Delivering ACT with sensitivity

Practitioners reported ACT should be communicated with sensitivity. According to practitioners, for clients, the notion of accepting and defusing from emotions, thoughts, and memories that pertain to their loved one, while reconnecting with value-directed action without their loved one, could be viewed as dismissive of the significance of the deceased.

*Helping you reconnect with your values and helping you distance yourself from certain thoughts when they’re not helpful, now that can come across the wrong way, I think, if you do it clumsily or too directly. The wrong way, I mean, I guess, dismissive.* (Pierre)

To avoid these processes being understood as an encouragement to move on, let go, or disregard internal experiences that concern their loved one, practitioners spent time and care introducing ACT components and exercises.

*There is a lot of explaining about the language. Sometimes when people hear the word ‘acceptance’ it’s like a red flag to a bull. Because they’re in so much pain. […] So, I find I have to do a lot of prep work with people, in the beginning, to say […] it’s not about just a be-quiet-and-get-over-it type thing. It’s something much more involved than that.* (Alba)

### Adapting ACT according to individual differences

Practitioners adapted ACT according to their clients’ age. Practitioners reported that children and young people often had an incomplete and/or inaccurate understanding of grief. Consequently, practitioners adapted ACT to include more psychoeducation to facilitate acceptance and value-based work.

*When I’m working with younger people, it’s trying to figure out how many pieces of the puzzle do they have; what they have been told by adults around them; and what meaning do they make from that? And is any of that interrupting their grieving process?* (Trisha)

*I’d usually do a lot of psychoeducation with kids on bereavement […] once they’ve got that level of information, then it’s quite easy to do things like [the ACT exercise] “choice points”.* (Alba)

To facilitate children and young people’s understanding of grief and increase the efficacy of therapy, practitioners used more interactive strategies rather than conversation with abstract models and language.

*Value-based work – particularly with a deck of values cards – is great for kids. They’re usually really good at, kind of, picking out what’s key to them [;…] they are quite used to conversations about the future, usually in terms of subjects or studying or jobs.* (Alba)

Alba, Hanna, and Samantha viewed older clients as having greater difficulty staying in contact with, accepting, and expressing distressing internal experiences. All three practitioners said this was due to generational cultural values that discourage the expression of emotion.

*Accepting internal experiences can be very, very challenging for people who have been raised with rigid expectations about emotional expression. And I find that that can be more true of older adults.* (Hanna)

To facilitate contact with distressing internal experiences, practitioners took greater care in explaining ACT and adapted their approaches. To bring older clients into the present, Alba used mindfulness exercises that were directed at experiences of external rather than internal phenomena (e.g. an object in the room). To encourage contact with internal experiences, Hanna modelled noticing and expressing her own experiences. Samantha would also spend more time facilitating the mindfulness processes with older clients.

*The stereotypes in older adult services around mental health and psychiatric care are very, very strong. And that can mean that you have to do a lot more work on the defusion side of things. There’s just more to unpick there. And similarly, things like mindfulness, kids are quite, sort of, familiar with it now […] quite open minded in terms of trying different things. Whereas you can try anything with an older adult, but it usually just takes a bit more selling […] a bit more, kind of, explanation in practice.* (Alba)

Practitioners adapted ACT metaphors and language to ensure these were understood and effective with clients, taking into account individual differences in education and cognitive ability.

*I try to be really thoughtful about ‘does this intervention that I’m offering land?’ […] I certainly adapt for cognitive ability […] if I’m going to be building a metaphor with someone who had limited education, I’m really going to be mindful that it is not my metaphor, it is their metaphor […] so they’re building the metaphor in a way that makes sense to them.* (Ophelia)

### Other interventions can be complementary

Practitioners typically offered bereavement support to clients without explicitly mentioning ACT. Nonetheless, most practitioners drew on ACT as their primary approach to bereavement support. However, they mentioned at least eight other interventions they sometimes used alongside it, including cognitive behavioural therapy, narrative therapy, schema therapy, compassion-focused therapy, dialectical behaviour therapy, behavioural activation therapy, art therapy, and exposure therapy. Practitioners stated that, since ACT focuses on general psychological flexibility, other interventions can often be complementary.

*I think ACT is so flexible that it can adapt to take on other models. But I think the way I adapt, in that respect, is always through that filter of ‘We’re not trying to get rid of [symptoms]; we’re always aiming for psychological flexibility’.* (Samantha)

When the bereavement was traumatic, several practitioners used trauma-focused interventions in conjunction with ACT. Trauma was usually related to violent or distressing end-of-life experiences. Techniques that were adapted from post-traumatic stress disorder (PTSD) treatments often used variations of imaginal exposure: whereby the client recalls traumatic memories surrounding the death in detail.

*I do exposure therapy […] I ask [clients] to tell me what happened in a specific way: to retell the story of a loss in a way that means that they can be open to experience difficult thoughts; in a way that they can recognise the feelings that come and try to make sense of it. So that’s not an ACT technique, but I think it fits quite well with it.* (Pierre)

## Discussion

Our findings suggest that practitioners use ACT to: (i) improve clients’ relationship with grief through acceptance and the creation of psychological space around distressing internal experiences; (ii) support clients to reconnect with their values after loss and draw on them to engage in value-directed action; (iii) provide flexible support that is sensitive to the significance of the deceased, the characteristics of the bereaved person, and their bereavement support needs.

Practitioners emphasised the value of ACT for managing overwhelming emotion and intrusive thoughts and feelings, alongside the importance of acceptance and values clarification as part of the therapeutic process. These findings align with Walker’s doctoral research [32] that found that ACT was perceived as helpful in addressing undesirable thoughts and feelings, while helping people adapt to life without the deceased by identifying a new purpose and place in society. In both studies, practitioners were open to tailoring ACT based on the individual needs of the client, including adapting metaphors and integrating ACT with other therapeutic approaches.

Our findings that practitioners viewed acceptance and mindfulness techniques as improving clients’ ability to cope with difficult thoughts and feelings resonates with the results of a feasibility trial of an ACT self-help booklet for bereavement support [31]. In that study, bereaved clients reported that booklet sections on experiential avoidance and mindfulness were particularly helpful. Together, these findings highlight the perceived value of ACT acceptance and mindfulness techniques to support the bereaved person to cope following loss.

In our study, practitioners viewed avoidance strategies as detrimental in their own right: they were exhausting, took up psychological space, and interfered with the natural healing process. This is consistent with studies that show avoidance is positively associated with bereavement-related distress in university students [40] and prolonged grief disorder [41–45]. ACT tools for tackling avoidance may have multiple benefits with regards to the intensity and duration of bereavement-related distress.

According to practitioners in this study, clarifying values and committing to value-directed action in the midst of grieving can improve client coping and well-being. This is consistent with two studies that found that value-directed action is negatively associated with bereavement-related distress in university students [40, 43]. Moreover, prolonged grief disorder is associated with avoidance of recreational, social, and occupational activities (known as depressive avoidance; 46–48). Practitioners in this study reported clarifying values and committing to value-directed action can address a loss of purpose, meaning, and identity following bereavement, and provide the energy and purpose to move forward. ACT may facilitate renewed engagement with activities that are important to the person, supporting the restoration-oriented coping described by the Dual Process Model [27] and mitigating some of the disruption associated with grieving.

Practitioners considered all ACT processes as core to bereavement support. This is consistent with a recent meta-analysis of mediational studies that found that the combined flexibility processes account for therapeutic effects of ACT [49]. Furthermore, in our study, practitioners reported that both the mindfulness processes, and the values clarity and committed action processes contributed to improvements in coping and well-being independently. This result is similar to that of a study that found various combinations of the flexibility processes led to improvements in people experiencing clinical levels of psychological distress, but the greatest improvements were achieved when all process where combined [50]. Further investigations into the benefits of prioritising the flexibility processes according to individual differences are merited.

Practitioners were careful to avoid acceptance, defusion, and values work being perceived as dismissive of the significance of a client’s loved one. Given the double-edged meaning of words like ‘acceptance’, such language could become a barrier to accessing interventions. Moreover, practitioners adapted ACT metaphors and language according to individual differences and when introducing mindfulness processes to older adults, as the latter were thought to have more difficulty staying in contact with, accepting, and expressing distressing internal experiences. Overall, to maximise engagement with ACT, practitioners reported sensitivity is needed when selecting the language and techniques used to deliver ACT for bereavement support.

In this study, practitioners found it beneficial to use other therapeutic interventions alongside ACT at times. This openness to combining therapeutic approaches was also reported in Walker’s doctoral research [32]. In our study, other interventions were often chosen because they targeted a specific issue. For example, for traumatic bereavement, practitioners sometimes used imaginal exposure techniques based on PTSD treatments (e.g. 51). Trauma psychoeducation and imaginal exposure can reduce distress when confronted with traumatic memories [52] and thereby also facilitate ACT’s focus on openness to experiencing (accepting) distress. Psychoeducation was also used alongside ACT, especially when supporting children and young people. A primary function of psychoeducation is to create a vocabulary for discussing thoughts and feelings relating to grief and loss [53]. This can be a helpful first step in recognising the distressing grief responses that ACT techniques address. These findings suggest ACT-based bereavement support may benefit from the inclusion of psychoeducation and targeted interventions from other therapeutic approaches.

### Strengths and limitations

To our knowledge, this is one of few studies on the use of ACT for bereavement support [31, 32, 54]. Its rich analysis of practitioners’ perspectives added novel insights into an array of ACT therapeutic practices and adaptations and how they may lead to improvements in coping and well-being. However, some limitations should be considered: (i) we relied on a convenience sample for pragmatic reasons and our inclusion criteria were fairly open, rendering it difficult to accurately judge the level of ACT expertise amongst participants; (ii) due to time constraints, interview transcripts were not returned to practitioners for review (however, both practitioners who provided feedback on the final draft article were positive and did not request amendments); (iii) practitioners’ opinions on the utility of ACT practices and outcomes may differ from those of clients [55]; (iv) practitioners used other therapies in conjunction with ACT, therefore, perceived improvements in their clients may have been due to other factors; (iv) practitioners in this study were all Western, white, and working in the UK or USA – results may not be transferable to other cultural, ethnic, or health and social care contexts [56].

### Implications for bereavement support

First, clinical and research work may benefit from retaining flexible protocols as practitioners found all ACT practices are useful but best tailored to individual needs. Second, protocols may need to be adapted to (a) ensure that techniques that foster acceptance, defusion, and values are not seen as dismissive of the individual’s loved one and (b) accommodate potential developmental, age-related, and cohort effects around language comprehension, recognition of grief-related experiences, and openness to emotional expression and mindfulness.

### Future directions for research

Future research should explore whether practitioners’ perspectives on the benefit of ACT for bereavement support are consistent with clients’ perspectives. Whether they extend to practitioners and clients from diverse cultural, ethnic, and national backgrounds should also be examined. Studies are also needed to investigate the effectiveness of ACT for bereavement support as a technique in its own right and used alongside other psychological interventions. Further investigations into adaptations to ACT language and techniques to accommodate bereavement-related sensitivity and individual differences across a range ages, cohorts, and cognitive abilities are also warranted.

## Conclusions

Practitioners reported ACT is used to improve coping and well-being for bereaved people by supporting them to create psychological space around distressing internal experiences and use that space to engage in value-directed action. While all ACT processes were considered core to bereavement support, practitioners stated they should be tailored to individual needs. Practitioners claimed ACT language and techniques should be used with sensitivity, adapting them to cognitive ability and age-related and cohort effects, while ensuring defusion, acceptance, and values work are not perceived as dismissive of the significance of the deceased. Finally, practitioners reported ACT for bereavement support may be enhanced by other therapeutic approaches. Practitioners viewed ACT as an effective bereavement support intervention; further research and intervention development is recommended.

### Electronic supplementary material

Below is the link to the electronic supplementary material.

Supplementary Material 1

## Data Availability

The datasets used and/or analyzed during the current study are available from the corresponding author on reasonable request.

## Abbreviations

ACT: acceptance and commitment therapy
CYP: children and young people
PTSD: post-traumatic stress disorder
UK: United Kingdom
USA: United States of America

## References

[1] Simon NM (2013). Treating complicated grief. JAMA.

[2] Worden JW (2008). Grief counseling and grief therapy: a handbook for the mental health practitioner.

[3] Stroebe M, Schut H (2010). The dual process model of coping with bereavement: a decade on. OMEGA-Journal Death Dying.

[4] Stroebe M, Schut H, Stroebe W (2007). Health outcomes of bereavement. Lancet.

[5] Zisook S, Shear K (2009). Grief and bereavement: what psychiatrists need to know. World Psychiatry.

[6] Bui E, Bui E (2018). Grief: from normal to pathological reactions. Clinical handbook of Bereavement and grief reactions.

[7] Zisook S, Iglewicz A, Avanzino J, Maglione J, Glorioso D, Zetumer S (2014). Bereavement: course, consequences, and care. Curr Psychiatry Rep.

[8] Arizmendi BJ, O’Connor MF (2015). What is normal in grief?. Aust Crit Care.

[9] Lobb EA, Kristjanson LJ, Aoun SM, Monterosso L, Halkett GKB, Davies A (2010). Predictors of complicated grief: a systematic review of empirical studies. Death Stud.

[10] Killikelly C, Maercker A (2017). Prolonged grief disorder for ICD-11: the primacy of clinical utility and international applicability. Eur J Psychotraumatol.

[11] Kersting A, Brähler E, Glaesmer H, Wagner B (2011). Prevalence of complicated grief in a representative population-based sample. J Affect Disord.

[12] Lundorff M, Holmgren H, Zachariae R, Farver-Vestergaard I, O’Connor M (2017). Prevalence of prolonged grief disorder in adult bereavement: a systematic review and meta-analysis. J Affect Disord.

[13] American Psychiatric Association (2013). Diagnostic and statistical manual of mental disorders: DSM-5.

[14] Aoun S, Breen L, O’Connor M, Rumbold B, Nordstrom C (2012). A public health approach to bereavement support services in palliative care. Aust N Z J Public Health.

[15] National Institute for Health and Care Excellence (NICE). Improving supportive and palliative care for adults with cancer. 2004. https://www.nice.org.uk/guidance/csg4 Accessed 19 May 2022.

[16] Savanta. Sue Ryder – Bereavement support survey. 2019. https://savanta.com/knowledge-centre/poll/sue-ryder-bereavement-support-survey/ Accessed June 6 2022.

[17] Hewison A, Zafar S, Efstathiou N (2020). Bereavement support in the UK – a rapid evidence assessment. Bereave Care.

[18] Keyes KM, Pratt C, Galea S, McLaughlin KA, Koenen KC, Shear MK (2014). The burden of loss: unexpected death of a loved one and psychiatric disorders across the life course in a national study. Am J Psychiatry.

[19] Hayes SC (2004). Acceptance and commitment therapy, relational frame theory, and the third wave of behavioral and cognitive therapies. Behav Ther.

[20] Hayes SC, Wilson KG, Gifford EV, Follette VM, Strosahl K (1996). Experiential avoidance and behavioral disorders: a functional dimensional approach to diagnosis and treatment. J Consult Clin Psychol.

[21] Hayes SC, Strosahl K, Wilson KG, Steven C, Hayes KD, Strosahl, Kelly G, Wilson. 2nd ed. New York: Guilford Press; 2012.

[22] Hayes SC, Pistorello J, Levin ME (2012). Acceptance and commitment therapy as a unified model of behavior change. Couns Psychol.

[23] Twohig MP (2012). Acceptance and commitment therapy: introduction. Cogn Behav Pract.

[24] Fletcher L, Hayes SC (2005). Relational frame theory, acceptance and commitment therapy, and a functional analytic definition of mindfulness. J Rational-Emotive Cognitive-Behavior Therapy.

[25] Harris R (2008). The happiness trap.

[26] Harris R. ACT made simple: an easy-to-read primer on acceptance and commitment therapy. New Harbinger; 2019.

[27] Schut MS, Henk (1999). The dual process model of coping with bereavement: rationale and description. Death Stud.

[28] Klass D, Silverman PR, Nickman SL (1996). Continuing bonds: new understandings of grief.

[29] Stroebe M, Schut H, Boerner K (2010). Continuing bonds in adaptation to bereavement: toward theoretical integration. Clin Psychol Rev.

[30] Root BL, Exline JJ (2014). The role of continuing bonds in coping with grief: overview and future directions. Death Stud.

[31] Davis EL, Deane FP, Lyons GC, Barclay GD, Bourne J, Connolly V (2020). Feasibility randomised controlled trial of a self-help acceptance and commitment therapy intervention for grief and psychological distress in carers of palliative care patients. J Health Psychol.

[32] Walker KA (2013). The experiences of therapists and bereaved clients of using an acceptance and commitment therapy.

[33] Jones K, Methley A, Boyle G, Garcia R, Vseteckova J (2021). A systematic review of the effectiveness of acceptance and commitment therapy for managing grief experienced by bereaved spouses or partners of adults who had received palliative care. Illn Crisis Loss.

[34] Gale NK, Heath G, Cameron E, Rashid S, Redwood S (2013). Using the framework method for the analysis of qualitative data in multi-disciplinary health research. BMC Med Res Methodol.

[35] QSR International Pty Ltd. NVivo. Version 12 Plus. 2018. https://www.qsrinternational.com/nvivo-qualitative-data-analysis-software/home2018.

[36] Tong A, Sainsbury P, Craig J (2007). Consolidated criteria for reporting qualitative research (COREQ): a 32-item checklist for interviews and focus groups. Int J Qual Health Care.

[37] Baillie L (2015). Promoting and evaluating scientific rigour in qualitative research. Nurs Stand.

[38] Hadi MA, José Closs S (2016). Ensuring rigour and trustworthiness of qualitative research in clinical pharmacy. Int J Clin Pharm.

[39] Clarke V, Braun V. Thematic analysis: a practical guide. Thematic Anal. 2021:1–100.

[40] Murrell AR, Jackson R, Lester EG, Hulsey T (2017). Psychological flexibility and resilience in parentally bereaved college students. OMEGA - J Death Dying.

[41] Boelen PA, Reijntjes A (2008). Measuring experiential avoidance: reliability and validity of the Dutch 9-item Acceptance and Action Questionnaire (AAQ). J Psychopathol Behav Assess.

[42] Boelen PA, van den Bout J, van den Hout MA (2010). A prospective examination of catastrophic misinterpretations and experiential avoidance in emotional distress following loss. J Nerv Ment Dis.

[43] Davis EL, Deane FP, Lyons GCB (2016). Prediction of individual differences in adjustment to loss: Acceptance and valued-living as critical appraisal and coping strengths. Death Stud.

[44] Eisma MC, Stroebe MS, Schut HAW, Stroebe W, Boelen PA, van den Bout J (2013). Avoidance processes mediate the relationship between rumination and symptoms of complicated grief and depression following loss. J Abnorm Psychol.

[45] Nam I (2016). Suicide bereavement and complicated grief: experiential avoidance as a mediating mechanism. J Loss Trauma.

[46] Boelen P, Van den Bout J (2010). Anxious and depressive avoidance and symptoms of prolonged grief, depression, and post-traumatic stress disorder. Physiol Belgica.

[47] Boelen PA (2012). Variables mediating the linkage between loss centrality and postloss psychopathology. J Nerv Ment Dis.

[48] Boelen PA, Eisma MC. Anxious and depressive avoidance behavior in post-loss psychopathology: a longitudinal study. Anxiety, Stress, & Coping. 2015;28(5):587–600. 10.1080/10615806.2015.1004054 25567154

[49] Stockton D, Kellett S, Berrios R, Sirois F, Wilkinson N, Miles G (2019). Identifying the underlying mechanisms of change during Acceptance and Commitment Therapy (ACT): a systematic review of contemporary mediation studies. Behav Cogn Psychother.

[50] Villatte JL, Vilardaga R, Villatte M, Plumb Vilardaga JC, Atkins DC, Hayes SC (2016). Acceptance and commitment therapy modules: differential impact on treatment processes and outcomes. Behav Res Ther.

[51] Foa E, Hembree EA, Rothbaum BO, Rauch S. Prolonged exposure therapy for PTSD: emotional processing of traumatic experiences - therapist guide. 2nd ed. Oxford University Press; 2019.

[52] Rauch SAM, Eftekhari A, Ruzek JI (2012). Review of exposure therapy: a gold standard for PTSD treatment. J Rehabilitation Res Dev.

[53] Kentor RA, Kaplow JB (2020). Supporting children and adolescents following parental bereavement: guidance for health-care professionals. Lancet Child Adolesc Health.

[54] Malmir T, Jafari H, Ramezanalzadeh Z, Heydari J (2017). Determining the effectiveness of Acceptance and Commitment Therapy (ACT) on life expectancy and anxiety among bereavedpPatients. Mater Sociomed.

[55] Walfish S, McAlister B, O’Donnell P, Lambert MJ (2012). An investigation of self-assessment bias in mental health providers. Psychol Rep.

[56] Houghton C, Casey D, Shaw D, Murphy K (2013). Rigour in qualitative case-study research. Nurse Res.

