# Supplementary material for: Practitioner perspectives on the use of acceptance and commitment therapy for bereavement support: a qualitative study
Source: BMC Palliat Care. 2024 Feb 28;23:59. doi: 10.1186/s12904-024-01390-x (PMC10900636; doi:10.1186/s12904-024-01390-x)
Supplement: Supplementary file 1 — Supplementary Material 1 [file 12904_2024_1390_MOESM1_ESM.docx]

**Interview questions (numbers) and prompts (letters)**

1. **Can you tell us the circumstances in which you have used ACT with people who are bereaved?**
   1. Can you describe your typical clients?
   2. Do you also work with children/adults/older people?
   3. How much time do you typically spend with your clients?
      1. length of sessions/number of sessions
      2. What factors influence this?
2. **How do you use ACT for bereavement support in practice?**
   1. Can you give me an example of how you use specific ACT components or processes?
   2. In what order would you introduce different aspects of ACT for bereavement support?
      1. What influences this?
   3. Are there specific methods or exercises that you think are particularly useful when supporting people who are grieving?
   4. Can you give some examples?
   5. To what extent do you adapt ACT to the person’s culture?
   6. To what extent do you adapt ACT according to relationship to the deceased?
   7. To what extent do you adapt ACT according to manner of death?
   8. To what extent do you adapt ACT according to the age of the client?
3. **In your experience, how does ACT lead to improvements for the person seeking support?**
   1. Can you give some examples?
   2. What elements of ACT are core to bereavement support?
4. **What are the challenges or obstacles you face using ACT with bereaved people?**
5. **If ACT is used alongside other bereavement support, can you describe how?**
   1. In which conditions would you use ACT?
   2. When would you use another intervention instead?
6. **To the second interviewer: is there anything you would like to ask?**
7. **To the participant: Is there anything that you would like to add that we haven’t discussed?**

**Participant Characteristics Questionnaire**

1. What is your current role(s)?
2. How long have you been in your current role (years)?
3. How long have you worked in a role providing bereavement support (years)?
4. Have you received formal training in ACT? (Please tick the suitable answer)

Yes [ ] No [ ]

1. If “yes”, please describe the training you have received (e.g. duration, qualification)
2. How long have you been using ACT to provide bereavement support (years)? Please note that, to participate, you must feel competent using ACT to provide bereavement support.
3. Do you use ACT for bereavement in support of (select one):
   - Children
   - Adults
   - Adults and children
4. Are you (select one):
   - Female
   - Male
   - Non-binary
   - Prefer not to say
   - Prefer to add my own description:
5. What age are you (select one)?
   - 18-24
   - 25-34
   - 35-44
   - 45-54
   - 55-64
   - 65+
   - Prefer not to say
6. What race/ethnicity do you identify with?

**Analytic Framework**

| Categories | Broad codes | Example Sub-codes | Example Excerpt |
| --- | --- | --- | --- |
| Flexibility processes | Acceptance | Control; avoidance; guilt | *Grief or death and dying, it's a key one that we don't have control over.* |
|  | Defusion | Suicidal thoughts; rumination, | *I would start with a very sort of gentle acceptance, defusion practice. Yeah. If the thoughts are the piece, that's really… In Virginia speak, ‘got their craw’* |
|  | Present moment awareness | Painful moments | *grief is a really tricky thing for being in the moment because the moment is so painful.* |
|  | Self-as-context | Perspective; sky and weather | *I think the skills and defusion awareness noticing and present moment and getting that distance, getting that perspective is it's just like gives people a way of being able to breathe because you can't make this stuff go away.* |
|  | Values clarity | Motivation; self-esteem | *You know, at some point, the values kind of propelled him to really generate some activities with his daughter.* |
|  | Committed action | n/a | *Folks have a lot of difficulty navigating the…I guess I'd call it organization like getting one’s mind organized to move forward. And so with those folks, I might begin even with the values and committed action of ‘Well, what's the next step?* |
| Exercises, Metaphors,  Psychoeducation | Exercises | Physicalisation; bullseye | *I'd quite often use a sort of bull's eye type worksheet with people and try to get a sense of where are they in terms of where they'd like to be* |
|  | Metaphors | Clipboard; Healing hand; curious child | *So if you imagine [defusion is] what your clipboard represents, and then when that happens, if you just imagine just putting it down in your lap, so you're not ignoring it, it's in front of you is, but what that enables you to do then is to see beyond that and connect, connect with the world, I suppose.* |
|  | Psychoeducation | Normalisation; familiarising, education | *Some people might come and they actually just want you to normalize grief a bit for them, they maybe don't understand what's going on.* |
| Generating change | Improvements | Less overwhelmed; flexibility | *Maybe someone just, you know, kind of wants to be less, less overwhelmed or less burdened. And so we're doing more defusion, we're doing more acceptance, that kind of thing* |
|  | Challenges | Language; older people | *I think one challenge that I have faced is me being overly bound to the concept or the theory of ACT and being really excited about it and trying to tell people ‘this isn't like CBT, we're not gonna fix your thoughts. We're gonna hold them gently, right?* |
|  | Core | All processes; mindfulness processes | *You know, [self-as-context], which I think is so important in ACT. It's sort of all the other skills there, the kind I see those as the foundational skills that then, on top of that, you can kind of start to introduce this idea of [self-as-context] that I am not my mind. I am not my thoughts.* |
|  | Other therapies |  |  |
| Adaptations | Age | Harder sell; stereotypes | *The stereotypes in older adult services around mental health and psychiatric care are very, very strong****.*** *And that that can mean that you have to do a lot more work on the defusion side of things.* |
|  | Culture | Religion; family culture | *I actively incorporate faith because I'm in a rural area of Virginia where folks have very significant ties to their religion.* |
|  | Manner of Death | Violent; COVID | *I do exposure therapy through narrative retelling more frequently when in ACT. And what that means is if I have the feeling that the loss is quite traumatic, when once they're ready, I ask them to tell me what happened in a specific way to retell the story of a loss* |
|  | Relationship to deceased | Child; long-term partnership | *Depending on how that's delivered, I think you can make quite a bit of resistance. And it can be hard, I think, especially with people that have been married for a really long time or if their child has died.* |
|  | Order | Client-led; no protocol | *I don't have a specific order very much. I'm very client led with it.* |
| Other | N/A | Anticipatory grief | *It might be that you have someone with a neurodegenerative condition where you know, the bereavement is coming. And ACT is really, really helpful for that as well.* |

**Example Framework Matrix**

*Analytic Framework Category: Generating Change*

| Practitioner | Broad Codes | | | |
| --- | --- | --- | --- | --- |
|  | ACT Core | Improvements | Challenges | Other therapies |
| Pierre | All processes are core and difficult to separate. But focuses on avoidance and fusion. But could be because of British culture. | Promotes psychological flexibility. People fight thoughts and feelings and are disengaged from values. ACT helps recontacting everything through understanding, mindfulness, metaphors. But you will never be the same. | Care not to interrupt natural grieving or be too directive. Care because ACT can come across as dismissive; defusion when someone is still making sense of things. | ACT is main therapy for grief. Also narrative reconstruction, mapping the problem, exposure therapy, but fits with ACT. |
| Alba | Techniques that keep clients in the present moment, giving them choice. Values to motivate action. Defusion to get unstuck from thoughts. | Emotion management with mindfulness. Then values work for action. | ACT language is tricky. Acceptance can be red flag to a bull. Older people have stereotypes regarding psychology and mindfulness. | Schema therapy for trauma. PTSD techniques for hyper arousal/vigilance. |
| Trisha | NA | Prevents avoidance by generating experiential and conceptual understanding. Gives people choice to into and away from grief. Separates from and takes power away from thought. Alternative is being consumed or overwhelmed | Reorientating people towards living again generates guilt. ACT can seem dismissive, especially if a child or long-term partner has died. | ACT techniques are part of integrative counselling |
| Ophelia | Usually mindfulness processes first, but sometimes values/committed action. Always dance around all processes. | ACT not glorified mindfulness. The purpose is important. Acceptance and defusion for being overwhelmed, burden. But without values not as effective; tries to do both. | Wanting to tell clients the theory behind ACT was problematic. To them, psychological “jibber jabber”. Stopped doing so. | ACT is main therapy. Compassion-focused therapy, attachment therapy, unconditional positive regard. DBT for suicidal and self-harming. |
| Angus | Grieving is a series of tasks to get to a place of acceptance. But then values are core; committing to a new life ahead. | We use ACT without realising it. Validation is acceptance. There is a clear place for ACT in bereavement support. | Language can be tricky. Clients need to be able to understand metaphors. Wouldn’t do it with Asperger’s syndrome. People need to be psychologically minded. | ACT supplementary to counselling training received as a social working. Uses continuing bonds and explains dual process model theory. |
| Samantha | Defusion takes precedence, but all processes are foundational. But they all contribute to perspective-taking (self-as-context) which is key. | Getting out of your head and into awareness. Mindfulness skills help with that: awareness and defusion provide relief; breath through the pain; emotion surf. Mindfulness, self-as-context, values, help you gain perspective and move forward. | Acceptance can come across in the wrong way for grievers. Emotional literacy is useful. ACT needs to be delivered with care in traumatic bereavement. ACT can be quite general, sometimes need targeted interventions. Some people can’t get past wanting to fix grief. Older people struggle being more open and aware. | ACT is main therapy. Exposure therapy, memory boxes, art therapy, DBT, attachment, CBT. But through ACT “filter”. |
| Stanley | NA | People like ACT and engage with it well. | No challenges. ACT is flexible, can deal with any problem. | Family therapy, death education. But everything is compatible with ACT. |
| Danielle | Allow grief to happen; don’t avoid it; it’s an important, exquisite experience. But then values propel people forward. ACT jumpstarts people. | Helps with understanding grief experientially. Mindfulness helps with letting pain come and go. Values creates willingness. Defusing from “will always” and “will never” thoughts. | Some people aren’t into mindfulness. Sometimes fusion so strong, people just want to tell their stories again and again. | ACT is go-to for grief. But uses anything useful. Doesn’t think about which therapy a technique belongs to. Anything that promotes flexibility is ACT congruent. |
| Hanna | All processes core. It’s about perspective taking (self-as-context). | Clients say clarity on values key. But all processes help people take perspective on themselves. Defusion helps with fear. | Some people reject mindfulness. Defusion can come across as dismissive. Traumatic bereavement requires care. Older people struggle with repressed emotions. | ACT is only therapy. |

*Note*: Each cell comprises a summary of the data. In NVivo, clicking on a summary retrieves all of the data coded with a broad code.

**Early Thematic Map**

**
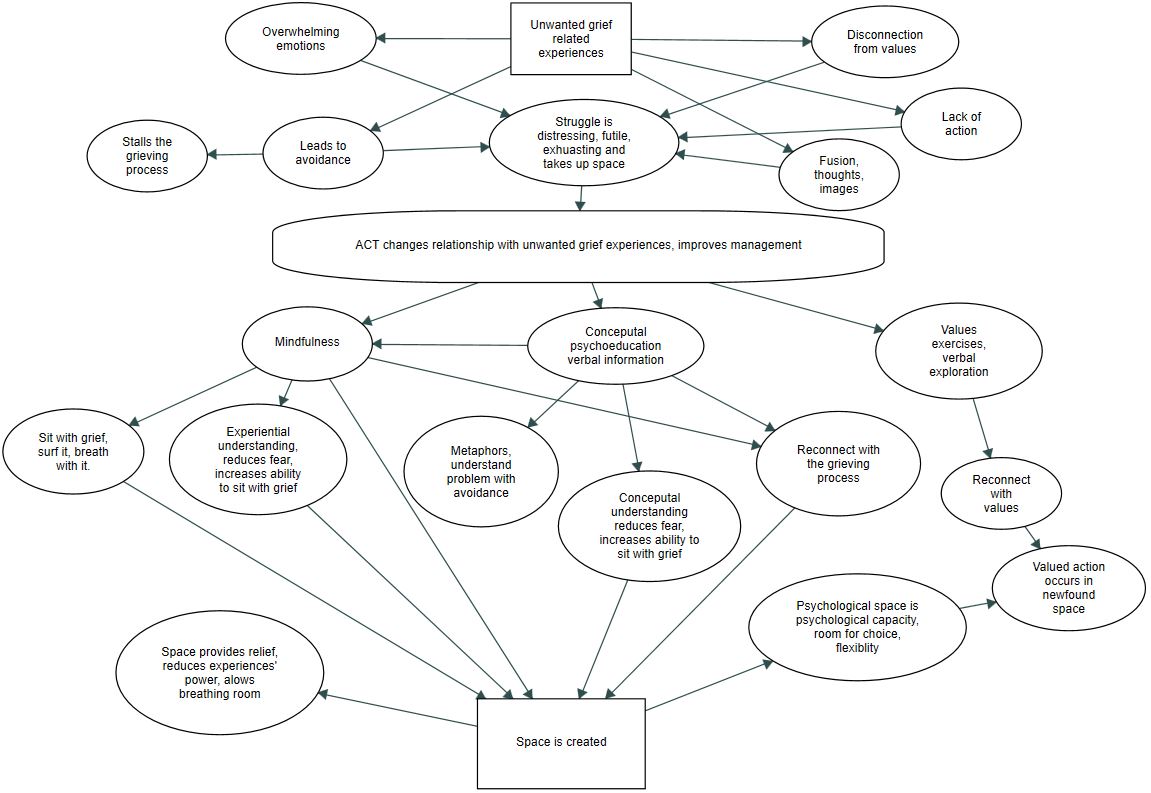
**
